# Association of Serum Vitamin D and Hematological Parameters with SARS-CoV-2 PCR Positivity: A Combined Biomarker Approach in Asymptomatic Children

**DOI:** 10.3390/ijms27104393

**Published:** 2026-05-14

**Authors:** Mehmet Almacioglu, Ipek Kocer, Demet Ari

**Affiliations:** 1 Department of Pediatrics, Faculty of Medicine, Sanko University, 27060 Gaziantep, Turkey; ipek.kocer1@gmail.com; 2 Department of Emergency Medicine, Faculty of Medicine, Kahramanmaraş Sütçü İmam University, 46100 Kahramanmaraş, Turkey; demetari@ksu.edu.tr

**Keywords:** Vitamin D, SARS-CoV-2, children, combined biomarker, risk stratification

## Abstract

Vitamin D has been implicated in immune modulation and susceptibility to respiratory infections, including COVID-19. However, data in asymptomatic pediatric populations, particularly those with household exposure, remain limited. This study aimed to investigate the association between serum vitamin D levels and hematological parameters with SARS-CoV-2 PCR positivity in asymptomatic children, and to evaluate their potential role in early risk stratification. This retrospective study included 127 asymptomatic children (aged 2–18 years) with confirmed household exposure to COVID-19. Participants were classified as PCR-positive (n = 74) or PCR-negative (n = 53). Serum 25(OH)D3 levels and hematological parameters were analyzed. Univariate and multivariable logistic regression analyses were performed to identify independent predictors. Receiver operating characteristic (ROC) curve analysis was used to assess discriminative performance, and a combined multimarker model was constructed. Serum vitamin D levels were significantly lower in PCR-positive children compared to PCR-negative children (17 ± 8 vs. 27 ± 11 ng/mL, p = 0.001). White blood cell (p = 0.002), platelet (p = 0.01), and neutrophil counts (p = 0.01) were significantly reduced, while basophil counts were higher in PCR-positive children (p = 0.02). In multivariable analysis, vitamin D (OR: 0.87, 95% CI: 0.82–0.93, p < 0.001), platelet (p = 0.02), neutrophil (p = 0.02), and basophil counts (p = 0.01) remained independent predictors. ROC analysis showed that vitamin D had moderate discriminative performance (AUC: 0.75, 95% CI: 0.67–0.83), while platelet (AUC: 0.64), neutrophil (AUC: 0.61), and basophil (AUC: 0.62) counts showed modest performance. The combined multimarker model demonstrated improved predictive ability (AUC: 0.80, 95% CI: 0.72–0.88), with sensitivity of 71.6% and specificity of 68.2%. Additionally, vitamin D deficiency was significantly more frequent in PCR-positive children (43% vs. 19%, p = 0.003). **Conclusions**: Lower vitamin D levels and associated hematological alterations are independently associated with SARS-CoV-2 PCR positivity in asymptomatic children. A combined biomarker approach may improve early risk stratification using simple and routinely available parameters. Further prospective studies are needed to validate these findings and clarify the role of vitamin D in preventive strategies.

## 1. Introduction

Since its emergence in late 2019, coronavirus disease 2019 (COVID-19) has significantly impacted global health systems. While pediatric cases are often asymptomatic or mild, children play a critical role in household transmission dynamics. Therefore, identifying biological factors associated with SARS-CoV-2 susceptibility in asymptomatic children is essential for improving early detection and risk assessment strategies [1].

Vitamin D is a molecule crucial for calcium-phosphorus metabolism and bone health. Emerging studies suggest that vitamin D may play a role in immune system regulation and the prevention of various diseases. In vitro studies provide evidence that vitamin D influences immune responses to viral and bacterial respiratory pathogens. A recent meta-analysis demonstrated that individuals with low 25(OH)D3 levels are at a higher risk of acute respiratory tract infections. Vitamin D appears to upregulate genes involved in immune responses to pathogens like *Streptococcus pneumoniae* [2,3,4,5,6]. A recent meta-analysis demonstrated that individuals with low 25(OH)D3 levels are at a higher risk of acute respiratory tract infections. More recently, a large-scale stratified meta-analysis by Jolliffe et al. further confirmed that vitamin D supplementation significantly reduces the risk of acute respiratory infections, particularly in individuals with baseline vitamin D deficiency, highlighting its immunoprotective role across different populations [7].

Laboratory studies indicate that respiratory epithelial cells can convert vitamin D to its active form, enhancing cytokine production in response to respiratory viruses [8]. Some reports suggest that individuals with low serum vitamin D levels might be at greater risk of COVID-19 infection or severe disease progression [9,10]. SARS-CoV-2, the causative agent of COVID-19, interacts with the renin–angiotensin system (RAS) through its spike (S) glycoprotein, binding to angiotensin-converting enzyme 2 (ACE2) [11,12]. RAS is expressed in the kidneys, blood vessels, lungs, intestines, heart, and immune cells, including monocytes and macrophages [13,14]. Vitamin D acts as a pro-hormone regulating RAS by suppressing its activity [14]. Dysregulated RAS and low vitamin D levels may lead to renal, cardiac, vascular, and immune complications in COVID-19 patients, contributing to cytokine storm syndrome [8,9,10,14].

Vitamin D mitigates multi-organ damage through various pathways. Recent focus has been placed on the immunomodulatory role of vitamin D [15,16]. The active form, 1,25-dihydroxy vitamin D (1,25(OH)2D3), suppresses T-helper 1 (Th1) cell proliferation and reduces inflammatory cytokines such as IL-6, IL-8, IL-12, and IL-17, thus inhibiting inflammation progression. It also downregulates TNF-α, nuclear factor-κB (NF-κB), IFN-γ, and IL-2 [17,18]. This regulation can suppress cytokine storms, preventing organ damage during severe COVID-19 infection. Alveolar epithelial cells can convert inactive vitamin D to its active form, promoting the expression of host defense genes like cathelicidin and defensin, which inhibit viral replication and reduce COVID-19 severity [19,20].

Vitamin D supplementation has been shown to prevent angiotensin II (Ang II) accumulation and suppress renin release, reducing inflammation and tissue damage in COVID-19 patients [10]. One study found that vitamin D exerts protective effects on alveolar epithelial cells, decreasing epithelial cell death and preventing acute respiratory distress syndrome (ARDS) in animal models [2]. Another study demonstrated that vitamin D supplementation reduces renin, ACE, and Ang II levels, increasing ACE2 expression and protecting against acute lung injury [5].

Epidemiological studies reveal that COVID-19 severity is greater in elderly and male patients, while children exhibit lower infection rates and milder symptoms [11,12]. Higher ACE2 expression in children and women compared to men and the elderly may explain this difference [11]. A study examining asymptomatic and mildly symptomatic COVID-19 patients found detectable levels of SARS-CoV-2-specific antibodies, but lower seroconversion rates in asymptomatic individuals [12].

Despite growing evidence on the role of vitamin D in COVID-19, data in pediatric populations—particularly among asymptomatic children with household exposure—remain limited. Most existing studies have focused on symptomatic or hospitalized patients, leaving a gap in understanding early or subclinical infection dynamics in children. Furthermore, the combined evaluation of vitamin D status with routine hematological parameters has not been sufficiently explored as a potential approach for identifying children at higher risk of SARS-CoV-2 PCR positivity following exposure.

It is well established that the immune response to respiratory infections in early life differs substantially from that of adults. In children, the innate immune system plays a more prominent and functionally dominant role, whereas the adaptive immune system is still in the process of maturation. Previous studies have demonstrated that innate immune responses, including epithelial barrier function, pattern recognition receptor signaling, and antimicrobial peptide production, are more active and critical in early life, particularly in the context of respiratory infections [21]. 

In addition, vitamin D has been shown to modulate innate immune responses by enhancing the expression of antimicrobial peptides such as cathelicidin and defensins, which are essential components of early host defense against respiratory pathogens [22]. This is particularly relevant in pediatric populations, where innate immunity constitutes the first line of defense and may partly explain differences in susceptibility, clinical presentation, and disease course of respiratory infections, including SARS-CoV-2.

This study aimed to investigate the association between serum vitamin D levels and hematological parameters with SARS-CoV-2 PCR positivity in asymptomatic children following household exposure, and to evaluate their potential role in early risk stratification. This study aimed to investigate the association between serum vitamin D levels and hematological parameters with SARS-CoV-2 PCR positivity in asymptomatic children following household exposure, and to evaluate their potential role in early risk stratification.

## 2. Results

Comparison of sociodemographic and laboratory parameters is shown in Table 1. The mean age of PCR-positive children (10.7 ± 4.9 years) was slightly higher than that of PCR-negative children (9.23 ± 5.41 years), although this difference was not statistically significant (p = 0.11). Gender distribution did not show significant variation between the groups (p = 0.48).

Vitamin D levels were significantly lower in PCR-positive children (17 ± 8 ng/mL) compared to PCR-negative children (27 ± 11 ng/mL) (p = 0.001). White blood cell (WBC) counts were also significantly lower in the PCR-positive group (6200 ± 2000 10^3^/μL) than in the PCR-negative group (7500 ± 2600 10^3^/μL) (p = 0.002). Platelet counts were notably lower in PCR-positive children (280.5 ± 77.2 10^3^/μL) compared to PCR-negative children (320 ± 92 10^3^/μL) (p = 0.009). Neutrophil counts were significantly reduced in the PCR-positive group (2900 ± 1200 10^3^/μL) compared to the PCR-negative group (4000 ± 2000 10^3^/μL) (p = 0.006). Basophil levels were higher in the PCR-positive group (68 ± 210 10^3^/μL) compared to the PCR-negative group (50 ± 237 10^3^/μL) (p = 0.020). Other parameters, such as red blood cell counts (p = 0.16), hematocrit (p = 0.14), hemoglobin (p = 0.01), mean platelet volume (p = 0.07), lymphocytes (p = 0.20), monocytes (p = 0.56), eosinophils (p = 0.54), neutrophil-to-lymphocyte ratio (NLR) (p = 0.69), and platelet-to-lymphocyte ratio (PLR) (p = 0.86), did not show statistically significant differences between the two groups (Table 1.).

Correlation analysis of vitamin D and other parameters in patients with PCR Positive is shown in Table 2. Serum vitamin D levels demonstrated a significant negative correlation with WBC (r = −0.29, p = 0.001), platelet counts (r = −0.24, p = 0.007), and neutrophil counts (r = −0.19, p = 0.04). A weak positive correlation was observed between vitamin D and basophil counts (r = 0.21, p = 0.02). No significant correlations were found between vitamin D levels and other clinical or hematological parameters (all p > 0.05) (Table 2.).

Univariate and multivariable logistic regression analyses were performed to identify factors associated with SARS-CoV-2 PCR positivity (Table 3). In univariate analysis, lower serum vitamin D levels were significantly associated with increased likelihood of PCR positivity (OR: 0.65, 95% CI: 0.56–0.75, p < 0.001). Similarly, lower platelet counts (OR: 0.79, 95% CI: 0.63–0.94, p = 0.008) and lower neutrophil counts (OR: 0.78, 95% CI: 0.65–0.93, p = 0.009) were also significantly associated with PCR positivity. In contrast, higher basophil counts were associated with an increased risk of PCR positivity (OR: 1.19, 95% CI: 1.01–1.39, p = 0.03), while white blood cell counts were not significantly associated with PCR results (p = 0.50). In multivariable logistic regression analysis, vitamin D, platelet, neutrophil, and basophil counts remained independently associated with PCR positivity (Table 3).

**Figure 1:**
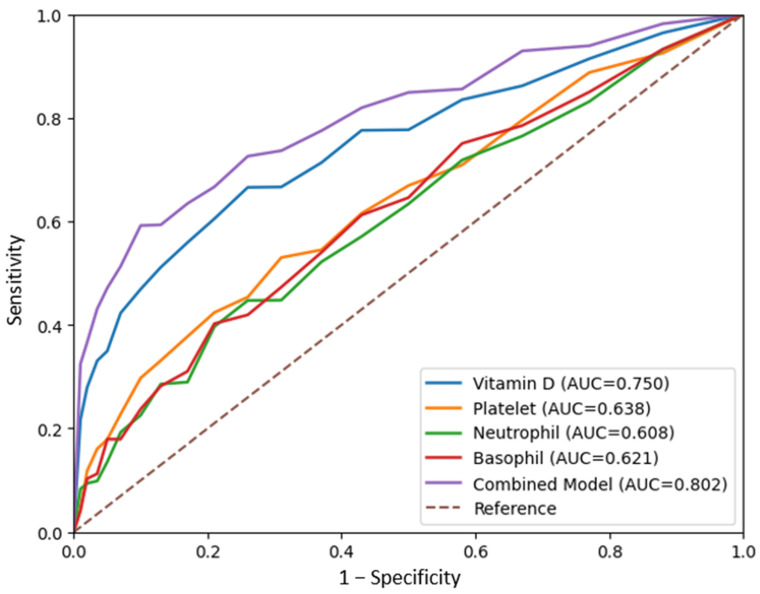
ROC curves of vitamin D, platelet, neutrophil, basophil, and combined model for predicting PCR positivity.

ROC analysis of the parameters used to predict PCR positivity is presented in Table 4. After correction for inverse association, vitamin D demonstrated moderate discriminative performance (AUC: 0.75, 95% CI: 0.67–0.84, p = 0.001). Platelet (AUC: 0.64), neutrophil (AUC: 0.61), and basophil (AUC: 0.62) counts showed modest predictive performance. A combined model including vitamin D, platelet, neutrophil, and basophil counts demonstrated improved discriminative performance compared to individual parameters (AUC: 0.80, 95% CI: 0.72–0.88, p < 0.001) (Table 4, Figure 1).

To further evaluate the combined effect of significant variables, a multivariable logistic regression model including vitamin D, platelet, neutrophil, and basophil counts was constructed as a multimarker model. In this model, all variables remained independently associated with PCR positivity, confirming the robustness of the combined model. Among these, vitamin D showed the strongest inverse association, while basophil counts demonstrated a positive association (Table 5).

To further evaluate the robustness of the findings, an additional multivariable logistic regression analysis adjusted for age, sex, and BMI was performed. After adjustment, serum vitamin D levels remained a significant independent predictor of SARS-CoV-2 PCR positivity (adjusted OR: 0.86, 95% CI: 0.80–0.92, p < 0.001). Platelet counts (adjusted OR: 0.99, 95% CI: 0.98–0.99, p = 0.02) and neutrophil counts (adjusted OR: 0.99, 95% CI: 0.99–1.00, p = 0.02) also retained their independent negative associations with PCR positivity. Basophil counts remained positively associated with PCR positivity (adjusted OR: 1.02, 95% CI: 1.01–1.03, p = 0.01). In contrast, age, sex, and BMI were not significantly associated with PCR positivity in the adjusted model (all p > 0.05) (Table 6).

Vitamin D levels were further analyzed by categorizing patients into deficiency (<12 ng/mL), insufficiency (12–20 ng/mL), and normal (>20 ng/mL) groups. The prevalence of vitamin D deficiency was significantly higher in PCR-positive children compared to PCR-negative children (43% vs. 19%). Conversely, normal vitamin D levels were more frequent in PCR-negative children (47% vs. 19%). The distribution of vitamin D categories differed significantly between groups (p = 0.003), indicating a higher likelihood of SARS-CoV-2 PCR positivity among children with lower vitamin D levels (Table 7).

## 3. Discussion

In the present study, we demonstrated that lower serum vitamin D levels were significantly associated with SARS-CoV-2 PCR positivity in asymptomatic children with household exposure. In addition, several hematological parameters, including decreased platelet and neutrophil counts and increased basophil levels, were independently associated with PCR positivity. Importantly, our findings showed that a combined multimarker model incorporating vitamin D and hematological parameters provided improved discriminative performance compared to individual markers alone, suggesting potential clinical utility for early risk stratification in pediatric populations.

Our findings regarding the inverse association between vitamin D levels and SARS-CoV-2 infection are consistent with previous studies. Reddy et al. demonstrated that lower circulating 25-hydroxyvitamin D levels were associated with significantly higher SARS-CoV-2 positivity rates in a large population-based analysis, supporting a potential role of vitamin D in susceptibility to infection [23]. Meltzer et al. reported that individuals with lower vitamin D levels had a higher likelihood of testing positive for COVID-19 [10]. Martineau et al. demonstrated in a large meta-analysis that vitamin D supplementation reduces the risk of acute respiratory tract infections, particularly in individuals with baseline deficiency [24]. Jolliffe et al. further confirmed the protective role of vitamin D against respiratory infections, emphasizing its role in enhancing innate immune responses [4]. Together, these findings support the hypothesis that vitamin D contributes to host defense mechanisms against respiratory pathogens, including SARS-CoV-2.

Although most previous studies have focused on adult populations, emerging pediatric data are generally in agreement with our results. Several studies have reported lower vitamin D levels in children with COVID-19 compared to controls, suggesting an association between vitamin D deficiency and infection susceptibility. However, our study extends the current literature by specifically focusing on asymptomatic children with household exposure, a subgroup that has been relatively underexplored. This is particularly important because asymptomatic children may play a significant role in transmission dynamics while remaining clinically unrecognized.

In addition to vitamin D, we identified significant associations between hematological parameters and PCR positivity, consistent with previous reports demonstrating that COVID-19 is associated with a broad spectrum of hematological abnormalities affecting immune and inflammatory pathways [25]. Henry et al. reported that thrombocytopenia is commonly observed in COVID-19 and may reflect disease severity [26]. Lippi et al. highlighted that alterations in hematological indices, including leukocyte parameters, are associated with adverse outcomes in COVID-19 patients [27]. Liu et al. also demonstrated that immune cell dysregulation plays a central role in disease progression [28]. While these studies primarily focused on hospitalized or severe cases, our findings suggest that even asymptomatic children may exhibit subtle but measurable hematological alterations, indicating early immune system involvement.

These findings are consistent with a growing body of evidence demonstrating that COVID-19 is associated with measurable alterations in hematological parameters, even in the absence of overt clinical symptoms. Previous studies have reported that viral infections, including SARS-CoV-2, can induce early changes in leukocyte subsets, platelet counts, and inflammatory markers through immune-mediated mechanisms. In particular, lymphocyte and neutrophil dynamics have been widely investigated as indicators of immune response and disease progression. While most existing studies have focused on hospitalized or symptomatic patients, our findings suggest that similar, albeit milder, hematological alterations may also be present in asymptomatic pediatric cases. This supports the concept that subclinical immune activation occurs even in the early or asymptomatic stages of infection. Moreover, the observed changes in platelet and neutrophil counts may reflect early immune system engagement and redistribution of immune cells in response to viral exposure. Taken together, these results indicate that routine hematological parameters may serve as accessible indicators of early immune response in pediatric SARS-CoV-2 infection, even in the absence of symptoms. This highlights the potential value of integrating hematological markers into multimodal risk assessment strategies.

A particularly notable finding of our study is the association between increased basophil counts and PCR positivity. Although the role of basophils in COVID-19, especially in pediatric populations, remains poorly understood, emerging evidence suggests that basophils may contribute to immune regulation beyond their traditional role in allergic responses [14]. Basophils are known to participate in Th2-mediated immune responses, cytokine secretion, and modulation of inflammatory pathways, which may influence the host response to viral infections. In the context of viral infections, basophils have been implicated in shaping immune responses through the release of interleukin-4 (IL-4) and other mediators, potentially affecting both innate and adaptive immunity. Furthermore, vitamin D has been reported to influence immune cell differentiation and inflammatory signaling pathways, raising the possibility that vitamin D deficiency may contribute to alterations in basophil activity or distribution. Given the limited data available, the observed association in our study may reflect an early immunological response pattern or a compensatory mechanism in asymptomatic infection. This finding highlights a potentially novel link between vitamin D status and basophil-mediated immune responses in SARS-CoV-2 infection, warranting further investigation in larger and mechanistic studies.

One of the key strengths of our study is the use of a combined multimarker model. While individual parameters demonstrated only modest discriminative ability, the combined model achieved improved performance, indicating that integrating metabolic and hematological markers may provide a more comprehensive assessment of infection risk. This approach aligns with recent trends in biomarker research, which emphasize the importance of multidimensional models rather than reliance on single parameters. The improved performance of the combined model in our study supports its potential utility in clinical risk stratification, particularly in resource-limited settings. Furthermore, the categorical analysis of vitamin D strengthened our findings by demonstrating that vitamin D deficiency was significantly more prevalent among PCR-positive children. This observation reinforces the clinical relevance of vitamin D status classification and suggests that deficiency thresholds may be useful in identifying at-risk pediatric populations. 

Despite these strengths, several limitations should be acknowledged. First, this was a retrospective, single-center study, which may limit the generalizability of the findings and introduce potential selection bias. Second, the relatively small sample size may reduce statistical power and affect the robustness of the observed associations. Third, although we adjusted for several variables, the possibility of residual confounding due to unmeasured factors cannot be excluded. In particular, the study period encompassed both winter and summer months, which may have influenced serum vitamin D levels. Although all participants were recruited from the same geographic region within a relatively short time frame, individual differences in sun exposure could not be fully controlled. In addition, although a conventional variable selection strategy was applied, alternative modeling approaches, including stepwise selection or multiple comparison corrections (e.g., Bonferroni adjustment), could be considered. However, given the exploratory nature of the study and the limited sample size, overly conservative correction methods may increase the risk of type II error and obscure clinically relevant associations. Despite these limitations, the use of multivariable analyses adjusted for age, sex, and BMI strengthens the robustness of our findings. In addition, the use of simple, routinely available laboratory parameters enhances the practical applicability of our findings in real-world clinical settings. The combined biomarker approach further strengthens the potential for early risk stratification using cost-effective and easily accessible tools. Furthermore, the focus on asymptomatic children represents a unique contribution to the literature, as this population remains underrepresented in COVID-19 research. In addition, several factors that may influence serum vitamin D levels were not fully assessed, including individual sunlight exposure, dietary intake, and prior supplementation history. These variables may contribute to inter-individual variability in vitamin D status and represent potential sources of bias. Although participants were recruited from the same geographic region within a relatively similar time frame, we could not account for these factors at an individual level, which may have influenced the observed associations. Future prospective longitudinal studies are needed to clarify the temporal and causal relationship between vitamin D status and susceptibility to respiratory infections.

## 4. Materials and Methods

### 4.1. Study Design and Participants

The study was designed as a descriptive, retrospective investigation. Ethical approval was obtained from the Non-Invasive Clinical Research Ethics Committee of SANKO University (Approval no: 2021/02, Date: 4 February 2021). This study included children aged 2 to 18 years who presented to the COVID-19 outpatient clinic at Sanko University Faculty of Medicine, Sani Konukoğlu Research Hospital, between February 2020 and July 2020. Participants were asymptomatic but had household contact with individuals diagnosed with COVID-19. The study included children who tested positive for SARS-CoV-2 by RT-PCR (n = 74) and those who tested negative (n = 53). The study period (February to July 2020) encompassed both winter and summer months, which may introduce seasonal variability in serum vitamin D levels due to differences in sunlight exposure. However, all participants were recruited from the same geographic region within a relatively short time frame, which may have partially mitigated the impact of seasonal variation across study groups.

**Figure 2:**
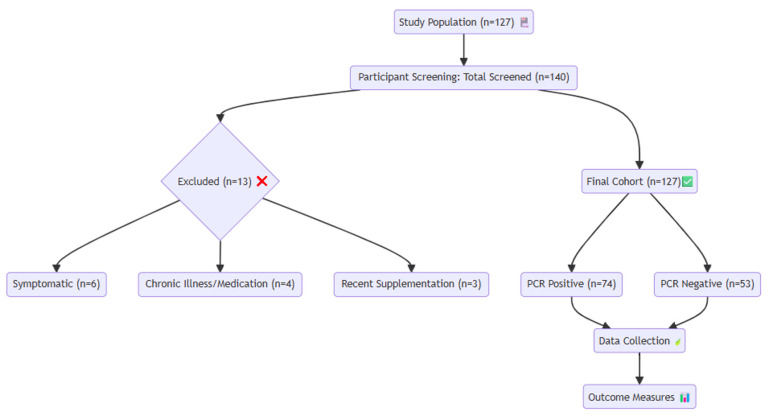
Flow diagram of inclusion, exclusion and extubation outcomes. Flowchart of the study population and study design. The participant icon represents the study population, the red cross mark icon indicates excluded participants, the green check mark represents the final cohort included in the analysis, the leaf icon indicates the data collection process, and the bar chart icon represents outcome measures and statistical evaluation.

Eligible participants had no symptoms during COVID-19 screening, no history of malnutrition or chronic disease based on anamnesis, physical examination, and laboratory findings, and had not received any regular medication, vitamin supplementation, or antibiotics in the past month. All participants lived in urban areas in apartment buildings. Children who did not meet these criteria were excluded from the study (Figure 2).

A symptom screening was performed for all participants, assessing for new-onset cough, shortness of breath, sore throat, nasal congestion, hoarseness, swallowing difficulty, loss of taste and smell, nausea, vomiting, abdominal pain, diarrhea, fatigue, weakness, headache, and fever. Data on gender, age, height, weight, complete blood count, biochemical parameters, and vitamin D levels were recorded from patient files.

Serum 25(OH)D3 concentration was used to assess vitamin D status. Measurements were performed using a Cobas 6000 analyzer series (Roche Diagnostics GmbH, Mannheim, Germany) with a Cobas e 601 module and a chemiluminescent immunoassay. Patients were categorized into three groups based on 25(OH)D3 levels: Group 1 (deficiency, <12 ng/mL), Group 2 (insufficiency, 12–20 ng/mL), and Group 3 (normal, >20 ng/mL). No participants exhibited vitamin D toxicity (>100 ng/mL).

SARS-CoV-2 RT-PCR tests were conducted on nasal swab samples obtained from asymptomatic household contacts, in accordance with Turkish COVID-19 guidelines.

### 4.2. Statistical Analysis

All statistical analyses were performed using IBM SPSS Statistics version 27.0 (IBM Corp., Armonk, NY, USA). The normality of continuous variables was assessed using the Shapiro–Wilk test. Normally distributed variables are presented as mean ± standard deviation (SD), while non-normally distributed variables are expressed as median (minimum–maximum). Comparisons between PCR-positive and PCR-negative groups were performed using the independent samples *t*-test or Mann–Whitney U test, as appropriate. Categorical variables were compared using the chi-square test. To identify factors associated with SARS-CoV-2 PCR positivity, univariate logistic regression analyses were initially performed. Variables with a p-value < 0.10 in univariate analysis were subsequently included in a multivariable logistic regression model to determine independent predictors. Results are presented as odds ratios (ORs) with 95% confidence intervals (CIs). Receiver operating characteristic (ROC) curve analysis was conducted to evaluate the discriminative performance of significant variables for predicting PCR positivity. The area under the curve (AUC) with 95% confidence intervals was calculated. For variables with AUC values below 0.5, inverse transformation was applied to ensure correct model interpretation. Optimal cut-off values were determined using the Youden index. Additionally, a combined model including significant variables was constructed to assess their joint predictive performance. Model discrimination was evaluated using AUC, and calibration was assessed using the Hosmer–Lemeshow goodness-of-fit test. To further ensure the robustness of the multivariable model, potential multicollinearity among candidate variables was assessed using correlation analysis and variance inflation factors (VIF). No significant multicollinearity was detected (all VIF values < 3), indicating that the included variables contributed independently to the model. Given the limited number of predictors relative to the sample size, correction for multiple comparisons (e.g., Bonferroni adjustment) was not applied to avoid excessive type II error, as recommended in exploratory clinical studies. A two-tailed p-value < 0.05 was considered statistically significant.

## 5. Conclusions

In conclusion, this study demonstrated that lower serum vitamin D levels are significantly associated with SARS-CoV-2 PCR positivity in asymptomatic children with household exposure. In addition to vitamin D, alterations in hematological parameters—particularly decreased neutrophil and platelet counts and increased basophil levels—were independently associated with PCR positivity. While the discriminative performance of individual biomarkers was modest, the combined multimarker model integrating vitamin D and hematological parameters showed improved predictive ability, suggesting that a composite approach may provide more reliable risk stratification. Furthermore, the higher prevalence of vitamin D deficiency among PCR-positive children highlights the potential clinical relevance of vitamin D status in identifying susceptible individuals. These findings support the hypothesis that vitamin D-related immune modulation and early hematological changes may play a role in SARS-CoV-2 susceptibility in pediatric populations. From a clinical perspective, the use of simple, routinely available biomarkers may facilitate early identification of at-risk children, particularly in settings with limited resources. However, given the retrospective design and relatively small sample size, further large-scale, prospective studies are needed to validate these findings. Although SARS-CoV-2 infection rates have declined compared to earlier phases of the pandemic, the clinical relevance of our findings extends beyond COVID-19. The immunomodulatory role of vitamin D and its interaction with hematological parameters may have broader implications for susceptibility to respiratory infections in pediatric populations. Therefore, future studies focusing on respiratory viral infections and immune-related biomarkers in children may further clarify the potential role of vitamin D in preventive and risk stratification strategies.

## Figures and Tables

**Table 1:** Comparison of sociodemographic and laboratory parameters.

Variable	PCR Negative (n = 53) Mean ± SD		PCR Positive (n = 74) Mean ± SD		p Value
Age (Year)	9	5	11	5	0.11
Gender					0.48
Female	28		34		
Male	25		40		
Weight (kg)	34	20	41	27	0.08
Height (cm)	127	33	137	30	0.01
Body Mass Index (BMI)	19	4	19	5	0.73
Vitamin D (ng/mL)	26.5	11.4	16.8	8.0	0.001
White Blood Cell (WBC, 10^3^/μL)	7500	2600	6200	2000	0.002
Red Blood Cell (RBC, 10^6^/μL)	4.9	0.5	5.0	0.5	0.16
Hematocrit (HTC, %)	39	4	40	3	0.14
Hemoglobin (HGB, g/dL)	13	2	13	1	0.10
Platelet (PLT, 10^3^/μL)	320	92	280	77	0.009
Mean Platelet Volume (MPV, fL)	8	1	8	1	0.07
Neutrophil (NEU, 10^3^/μL)	3700	2200	2854	1246	0.006
Lymphocyte (LYM, 10^3^/μL)	3145	1563	2801	1433	0.20
Monocyte (MON, 10^3^/μL)	643	305	614	238	0.56
Eosinophil (EO, 10^3^/μL)	140	1720	120	500	0.54
Basophil (BAS, 10^3^/μL)	50	237	68	210	0.020
Neutrophil-to-Lymphocyte Ratio (NLR)	1.2	20.2	1.0	5.8	0.69
Platelet-to-Lymphocyte Ratio (PLR)	105	677	113	392	0.86

**Table 2:** Correlation analysis of vitamin D and other parameters in patients with PCR Positive.

Variables	PCR Positive Correlation Coefficient (r), p Value
Age (Year)	r = 0.14, p = 0.11
Gender (Male)	r = 0.06, p = 0.45
Weight (kg)	r = 0.15, p = 0.08
Height (m)	r = 0.15, p = 0.08
Body Mass Index (BMI)	r = 0.03, p = 0.78
Vitamin D (ng/mL)	r = −0.47 **, p < 0.001
White Blood Cell (WBC, 10^3^/μL)	r = −0.28 **, p = 0.001
Red Blood Cell (RBC, 10^6^/μL)	r = 0.14, p = 0.11
Hematocrit (HTC, %)	r = 0.15, p = 0.10
Hemoglobin (HGB, g/dL)	r = 0.15, p = 0.09
Mean Corpuscular Volume (MCV, fL)	r = −0.07, p = 0.43
Platelet (PLT, 10^3^/μL)	r = −0.23 **, p = 0.007
Mean Platelet Volume (MPV, fL)	r = −0.17, p = 0.05
Neutrophil (NEU, 10^3^/μL)	r = −0.18 *, p = 0.04
Lymphocyte (LYM, 10^3^/μL)	r = −0.13, p = 0.14
Monocyte (MON, 10^3^/μL)	r = 0.01, p = 0.91
Eosinophil (EO, 10^3^/μL)	r = −0.05, p = 0.54
Basophil (BAS, 10^3^/μL)	r = 0.21 *, p = 0.02
Neutrophil-to-Lymphocyte Ratio (NLR)	r = −0.04, p = 0.69
Platelet-to-Lymphocyte Ratio (PLR)	r = 0.016, p = 0.86

* p < 0.05 was considered statistically significant. ** p < 0.001 was considered highly statistically significant.

**Table 3:** Logistic regression analysis of factors associated with SARS-CoV-2 PCR positivity.

Variables	Univariate Analysis OR (95% CI)	p Value	Multivariable Analysis OR (95% CI)	p Value
Vitamin D (ng/mL)	0.65 (0.56–0.75)	<0.001	0.87 (0.82–0.93)	<0.001
White Blood Cell (WBC)	0.93 (0.80–1.06)	0.50	1.00 (0.99–1.01)	0.27
Platelet (PLT)	0.79 (0.63–0.94)	0.01	0.99 (0.98–0.99)	0.02
Neutrophil (NEU)	0.78 (0.65–0.93)	0.01	0.99 (0.99–1.00)	0.02
Basophil (BAS)	1.19 (1.01–1.39)	0.03	1.02 (1.01–1.03)	0.01

OR, odds ratio; CI, confidence interval. Variables with p < 0.10 in univariate analysis were included in the multivariable model.

**Table 4:** ROC analysis of the parameters used to predict PCR Positivity.

Parameters	Cut-Off	Sensitivity (%)	Specificity (%)	AUC (95% CI)	p Value
Vitamin D (ng/mL) *	<16.5	58.1	61.3	0.75 (0.66–0.84)	0.001
Platelet (PLT, 10^3^/μL)	<269.5	55.4	59.6	0.64 (0.54–0.74)	0.005
Neutrophil (NEU, 10^3^/μL)	<2584.5	53.8	57.9	0.61 (0.51–0.71)	0.037
Basophil (BAS, 10^3^/μL)	>67.7	64.9	62.7	0.62 (0.52–0.72)	0.018
Combined Model (Vit D + PLT + NEU + BAS)	−	71.6	68.2	0.80 (0.72–0.88)	<0.001

* p < 0.05 was considered statistically significant.

**Table 5:** Multivariable logistic regression analysis of the combined multimarker model.

Variables	OR (95% CI)	p Value
Vitamin D (ng/mL)	0.87 (0.82–0.93)	<0.001
Platelet (PLT)	0.99 (0.98–0.99)	0.02
Neutrophil (NEU)	0.99 (0.99–1.00)	0.02
Basophil (BAS)	1.02 (1.01–1.03)	0.01

**Table 6:** Multivariable logistic regression analysis adjusted for age, sex, and BMI.

Variables	Adjusted OR (95% CI)	p Value
Vitamin D (ng/mL)	0.86 (0.80–0.92)	<0.001
Platelet (PLT)	0.99 (0.98–0.99)	0.02
Neutrophil (NEU)	0.99 (0.99–1.00)	0.02
Basophil (BAS)	1.02 (1.01–1.03)	0.01
Age (years)	1.03 (0.97–1.08)	0.31
Sex (male)	1.12 (0.65–1.93)	0.67
BMI (kg/m^2^)	1.01 (0.94–1.08)	0.74

**Table 7:** Association between vitamin D categories and SARS-CoV-2 PCR positivity.

Vitamin D Category	PCR Negative (n = 53)	PCR Positive (n = 74)	p Value
Deficiency (<12 ng/mL)	10 (19%)	32 (43%)	
Insufficiency (12–20 ng/mL)	18 (34%)	28 (38%)	
Normal (>20 ng/mL)	25 (47%)	14 (19%)	0.003

## Data Availability

The original contributions presented in this study are included in the article. Further inquiries can be directed to the corresponding author.

## Abbreviations

ACE2	Angiotensin-Converting Enzyme 2
ARDS	Acute Respiratory Distress Syndrome
AUC	Area Under the Curve
BAS	Basophil
BMI	Body Mass Index
CI	Confidence Interval
COVID-19	Coronavirus Disease 2019
HGB	Hemoglobin
HTC	Hematocrit
IL	Interleukin
MPV	Mean Platelet Volume
MCV	Mean Corpuscular Volume
MON	Monocyte
NEU	Neutrophil
NLR	Neutrophil-to-Lymphocyte Ratio
PCR	Polymerase Chain Reaction
PLR	Platelet-to-Lymphocyte Ratio
PLT	Platelet
RAS	Renin–Angiotensin System
RBC	Red Blood Cell
ROC	Receiver Operating Characteristic
RT-PCR	Reverse Transcription Polymerase Chain Reaction
SARS-CoV-2	Severe Acute Respiratory Syndrome Coronavirus 2
SD	Standard Deviation
Th1	T-helper 1
TNF-α	Tumor Necrosis Factor Alpha
WBC	White Blood Cell
WHO	World Health Organization
